# Rising living alone among the elderly in Iran: prevalence and associated factors

**DOI:** 10.1186/s12877-022-03309-8

**Published:** 2022-07-26

**Authors:** Mehri Shams Ghahfarokhi

**Affiliations:** Department of Social Sciences, University of Isfahan, Isfahan, Iran

**Keywords:** Living alone, Solo living, Elderly, Prevalence, Living arrangements

## Abstract

**Background:**

This study first examines the pattern and trend of elderly living alone during the last five censuses in Iran. Then, after describing the characteristics of the elderly who live alone, it determines how social, economic, and demographic characteristics relate to the solo living of the elderly.

**Methods:**

The data for people aged 60 and above are from two sources, including the aggregate data from five successive Iranian censuses and the individual data of 2% of the 2016 Iranian census. To determine the relative importance of variables such as sex, age, education, and activity status of the elderly, a set of logistic regression models using STATA software has been used for individual data analysis of the 2016 census.

**Results:**

The proportion of older people living alone in 1986, 1996, 2006, 2011, and 2016 was 9.1, 9.0, 10.9, 14.5, and 14.9%, respectively. City residents are less likely to live alone than villagers, and women are more likely to live alone than men. The odds of living solo among Illiterate, Primary school, Secondary & High school and Holding a high school diploma elderly higher than those with university education. Being a student and homemaker increases solo living relative to employees, but pensioners reduce solo living relative to employees. Moreover, the odds of solo living of the elderly in the early and middle stages of old age is less than in late old age. Also, the variables included in the analysis explained 16% of the variation in solo living.

**Conclusion:**

The prevalence of elderly solo living is increasing. And this increase continues due to the fundamental decline in childbearing, changes in family structure, and the effects of culture and tradition. Also, due to the rise in life expectancy, which increases the number of years of life with the disease, and disability, the lack of relief organizations will create more difficult conditions for the older people living alone.

## Background

Population aging is happening all over the world. Virtually every country in the world is expected to experience a significant rise in the proportion of people aged 60 and over between 2017 and 2050. Worldwide, the elderly made up about 13% of the population in 2017, and their share of the world's population is expected to increase to 21% by 2050 and 28% by the end of the twenty-first century. Aging is associated with widespread socio-economic changes occurring around the world. Rising income and education levels, advancing gender equality, empowering women, improving public health through having access to reproductive and sexual health care and medical technologies, globalization, urbanization, and migration are among the emerging demographic issues. Each of these evolutions changes the living arrangement [1]. The living arrangement is an essential issue in the aging discourse showing familial and non-familial relationships with those who cohabit with the elderly [2]; it also provides immediate social care and support for vulnerable elderly [3]. One type of living arrangement is solo living, meaning that the elderly live alone in the household. There have been many studies about this aspect of living arrangements in the world [4–11], but few studies in Iran were done, which is also general and was before the last decade, but this situation has begun to change. This article aims to fill this gap in the literature on the elderly living alone [12–14]. Since such knowledge and evidence about the lives of the elderly can strengthen the capacity to make decisions, design, plan, and formulate evidence-based policies in the country, the issue is important.

The number of older people living alone is increasing day by day. Solo living is a serious risk factor for ill-health in the elderly [8, 12–16]. Most studies have shown that living alone leads to poor mental health [17–19], depression [11, 20, 21], lower happiness [22], insufficient consumption of fruits and vegetables [23], higher mortality rate [24], and feeling to be alone [25]. According to the United Nations (2017), among the 143 countries or regions studied, the average percentage of people aged 60 and over who lived alone was 12%. Estimates of solo living ranged from the lowest of 1% in Afghanistan and Pakistan to the highest of 34% in Lithuania. Half of the countries have a solo living ratio between 7 and 21% [1].

The living arrangement is determined by demographic, economic, and cultural factors. First, the demographic factor forms the bedrock of common accommodation opportunities. Communities with higher fertility rates typically have larger families than communities with low fertility. Increased life expectancy improves the likelihood of intergenerational cohabitation. Second, economic and cultural factors are influential in realizing these demographic opportunities for cohabitation [26]. Therefore, in this article, variables such as sex, age, education, and activity status of the elderly have been considered.

This paper examines the prevalence of living alone during the last five censuses, then describes the characteristics of the elderly living alone, and finally investigates the extent to which social, economic, and demographic factors are related to living alone among the elderly. These findings not only provide information about the dynamics of living arrangements but also provide further evidence of changes in family life in Iran.

## Data and methods

### Study population

The data for people aged 60 and above are from two sources, including the aggregate data from five successive Iranian censuses (1986, 1996, 2006, 2011, and 2016) [27] and the individual data of 2% (147,547) of the 2016 Iranian census [28] (The Statistical Center of Iran publishes only 2% of the raw data of the Population and Housing Census).

### Statistical analysis

To identify the solo living patterns of the elderly, their sex, age, education, and activity status have been studied. Census aggregate data have provided information only on the age and sex of the elderly. Therefore, other variables (e.g., education and activity status) were extracted from individual data of the 2016 census. This limitation caused the study to examine changes in the age and sex composition of the elderly over time. Other variables are compared between sole and non- sole older persons. An analysis of two groups in a given year provides a better understanding of the differences (for example, 2016). Finally, a set of logistic regression models has been used to investigate the relative importance of different correlates of solo living for individual data of the 2016 census. The odd ratios with 95% confidence intervals (95% CI) were calculated. All statistical analyses were performed using Stata V. 12 software [29]. To understand how the effect is different in age groups of the elderly, logistic regression models have been estimated according to the age of the elderly. The age groups show the early stages of old age (60–69 years), middle old age (70–79 years), and late old age (80 years and older).

### Selection of covariates

The control variables selection procedure starts with a univariate analysis of each variable. Variables with a significant univariate test at some arbitrary level are selected as candidates for the multivariate analysis. It is based on a p-value cutoff point of 0.25. The cutoff value of 0.25 is supported by the literature [30–32]. Therefore, the variables of the place of residence, sex, age, education, and activity status of the elderly were considered as control variables.

### Ethical considerations

Much of social science research involves the conduct of surveys that do not seek to perform any invasive procedures on the human body directly. It is for this reason that much of this research passes under the category of minimum risk. Such a categorization facilitates an expedited review that is quick, simple and does not call for a full ethical review that much of medical research is subject to; this is true in settings across most countries, including Iran. Iranian censuses are guided by four major principles of ethical research that are relevant for protecting human subjects viz, the principles of autonomy, non-maleficence, beneficence, and justice.

## Results

Table 1 displays the number and proportion of older people living alone by sex and place of residence over the past three decades. Nationwide, in 1986, the percentage of older people living alone was about 9.1% (243981). In 1996, it decreased slightly and reached 9% (355610). In 2006, it came to 10.9% (555801). In 2011 and 2016, it reached 14.5% (889940) and 14.9% (1102241), respectively. The highest rise was between 2006 and 2011, which increased by about 33%. Living alone is significantly growing in urban and rural areas. Still, the most remarkable difference between rural and urban areas was observed in 2016, so 14.5% (774908) of urban elderly and 16% (326700) of rural elderly live alone. There is a significant growth between men and women in solo living during all years of the census. Still, the most remarkable difference can be seen in 2011 and 2016, with 5% of men versus 23.6% of women and 5.5% of men vs. 24.1% of women, respectively. The same pattern is kept for the difference between the sexes for urban and rural areas.

**Table 1:** Number and percentage of sole persons aged 60 years or over by sex and place of residence, Iran, 1986–2016

Area	Sex	1986	1996	2006	2011	2016
**Whole country**	**Males**	54,443 (3.8)	78,226 (3.7)	102,858 (3.9)	150,926 (5.0)	200,702 (5.5)
	**Females**	189,538 (15.0)	277,374 (15.1)	452,943 (18.4)	739,014 (23.6)	901,539 (24.1)
	**Total**	243,981(9.1)	355,610 (9.0)	555,801(10.9)	889,940 (14.5)	1,102,241(14.9)
**Urban**	**Males**	25,493 (3.6)	41,743 (3.5)	63,156 (3.8)	99,534 (4.8)	133,825 (5.0)
	**Females**	97,426 (14.3)	161,186 (15.0)	302,326 (19.0)	517,589 (24.0)	641,083 (23.8)
	**Total**	122,919 (8.9)	202,929 (9.0)	365,482 (11.2)	617,123 (14.6)	774,908 (14.5)
**Rural**	**Males**	28,759 (4.1)	36,346 (3.8)	39,600 (4.1)	51,317 (5.4)	66,477 (6.7)
	**Females**	91,576 (15.9)	115,802 (15.2)	150,443 (17.3)	221,217 (22.7)	260,223 (24.8)
	**Total**	120,335 (9.4)	152,148 (8.9)	190,043 (10.4)	272,534 (14.2)	326,700 (16.0)

Each year, the number of sole older persons is divided by the total number of persons aged 60 years or over multiplied by 100

**Fig. 1:**
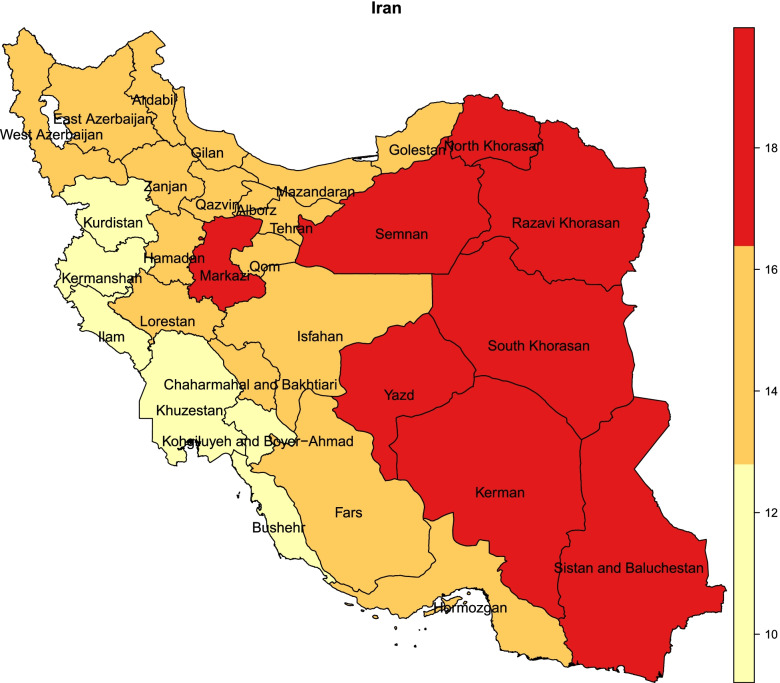
Map of Iran's geographical distribution according to the prevalence of solo living in the elderly, 2016

The prevalence of solo living in the elderly in Iran by province is shown in Fig. 1. The highest rate of solo living occurred in the provinces of South Khorasan (20%), Sistan and Baluchestan (19.7%), Semnan (18.7%), North Khorasan (18.4%), Yazd (18.2%), and provinces of Kohgiluyeh and Boyer-Ahmad (9.2%), Ilam (10.3%), Khuzestan (10.9%), Bushehr (11.2%) and Kurdistan (12.2%) had the lowest solo living rates. According to this geographical distribution map, which shows the solo living in the three groups, the eastern provinces of Iran had more solitary life than the western provinces (Fig. 1).

As displayed in Table 2, the proportion of older people living alone increases with age (30% of sole older people are aged 80 years and older*).* In the whole country, 15.55% of the illiterate elderly, 10.63% of the elderly with primary education, 8.39% of the elderly with secondary education, 8.51% of the elderly with high school diploma, and 7.89% of the elderly with higher education live alone. It is observed that 8.87% of the employed elderly, 6.53% of the elderly seeking work, 17.59% of undergraduate elderly, 19.52% of the homemakers, 12.77% of the pensioners, 14.21% of the elderly doing other activities live alone.

**Table 2:** Number and percentage of sole and non-sole older person by age composition, education, and activity status, Iran, 2016

Variable	Sole older person			Non-sole older person		
	**Males** (*n* = 4020)	**Females** (*n* = 17,904)	**Total** (*n* = 21,924)	**Males** (*n* = 68,732)	**Females** (*n* = 56,891)	**Total** (*n* = 125,623)
**place of residence**						
Urban (*n* = 106,785)	5.05 (17.27)	23.72 (82.73)	14.48 (100)	94.95 (54.95)	76.28 (45.05)	85.52 (100)
Rural (*n* = 40,762)	6.78 (20.88)	24.51 (79.12)	15.86 (100)	93.22 (54.08)	75.49 (45.92)	84.14 (100)
**Age (years)**						
60–69 (*n* = 84,985)	3.05 (16.88)	14.02 (83.12)	8.72 (100)	96.95 (51.31)	85.98 (48.69)	91.28 (100)
70–79 (*n* = 41,137)	5.71 (14.41)	33.33 (85.59)	19.64 (100)	94.29 (58.15)	66.67 (41.85)	80.36 (100)
≥ 80 (*n* = 21,425)	14.19 (24.95)	47.73 (75.05)	30.03 (100)	85.81 (64.73)	52.27 (35.27)	69.97 (100)
**Education**						
Illiterate (*n* = 79,094)	7.49 (14.41)	26.82 (85.59)	15.55 (100)	92.51 (43.26)	73.18 (56.74)	80.45 (100)
Primary school (*n* = 33,790)	4.23 (23.50)	19.88 (76.50)	10.63 (100)	95.77 (63.35)	80.12 (36.65)	89.37 (100)
Secondary & High school (*n* = 11,638)	3.33 (25.08)	17.08 (74.92)	8.39 (100)	96.67 (66.66)	82.92 (33.34)	91.61 (100)
Holding a high school diploma (*n* = 12,897)	4.14 (30.15)	15.67 (69.85)	8.51 (100)	95.86 (65.02)	84.33 (34.98)	91.49 (100)
University (*n* = 10,128)	4.85 (46.56)	17.34 (53.44)	7.89 (100)	95.15 (78.18)	82.66 (21.82)	92.11 (100)
**Activity status**						
Employed (*n* = 26,413)	4.84 (45.56)	29.18 (54.44)	8.87 (100)	95.16 (87.15)	70.82 (12.85)	91.13 (100)
Seeking work (*n* = 643)	5.05 (66.67)	15.73 (33.33)	6.53 (100)	94.95 (87.52)	84.27 (12.48)	93.47 (100)
Student (*n* = 108)	10.39 (42.11)	35.49 (57.89)	17.59 (100)	89.61 (77.53)	64.52 (22.47)	82.41 (100)
Homemaker (*n* = 57,337)	18.30 (1.15)	19.54 (98.85)	19.52 (100)	81.70 (1.25)	80.46 (98.75)	80.48 (100)
Income recipient (*n* = 43,816)	4.72 (29.17)	42.85 (70.83)	12.77 (100)	95.28 (86.17)	57.15 (13.83)	87.23 (100)
Other (*n* = 19,230)	7.81 (42.30)	35.62 (57.70)	14.21 (100)	92.19 (82.72)	64.38 (17.28)	85.79 (100)

Homemaker is not referring to a paid position; it refers to a person who does household chores;

Income recipients are persons receiving a pension or retirement benefits

The difference between the total and sum of parts is due to the persons whose activity status was not stated

Table 3 displays the results of logistic regression analysis for the effect of different correlates of solo living. The second column indicates the unadjusted odds ratios, which provide the impact of each variable without considering the effect of the other variables. The third column shows the estimated adjusted odds ratio based on the full model (including all variables). Columns 4, 5, and 6 include the results of the early old age (60–69 years), middle old age (70–79 years), and late old age (80 years and above), respectively.

**Table 3:** Results of logistic regression analysis of correlates of solo living by age composition, education, and activity status, Iran, 2016

**Variables**	** Unadjusted** **odds ratios** (95% CI)	** Adjusted** **odds ratios** (95% CI)	** Adjusted** **odds ratios (ages 60–69)** (95% CI)	** Adjusted** **odds ratios (ages 70–79)** (95% CI)	**Adjusted** **odds ratios (ages 80 +)** (95% CI)
**place of residence**					
Urban	0.90(0.87–0.93)^***^	0.97(0.93–1.01) ^NS^	1.08(1.01–1.14)^*^	0.97(0.92–1.04)^***^	0.84(0.78–0.90)^***^
Rural	Ref	Ref	Ref	Ref	Ref
**sex**					
Male	0.19(0.18–0.19)^***^	0.10(0.10–0.10)^***^	0.10(0.09–0.11)^***^	0.08(0.07–0.08)^***^	0.15(0.14–0.16)^***^
Female	Ref	Ref	Ref	Ref	Ref
**Education**					
Illiterate	2.84(2.63–3.06)^***^	1.62 (1.49–1.77)^***^	1.88 (1.68–2.10)^***^	1.70 (1.45–1.99)^***^	1.47 (1.14–1.89)^***^
Primary school	1.39(1.28–1.50)^***^	1.35 (1.24–1.48)^***^	1.40 (1.25–1.57)^***^	1.46(1.23–1.72)^***^	1.52 (1.17–1.97)^***^
Secondary & High school	1.07(0.97–1.18) ^NS^	1.14(1.03–1.27)^**^	1.18(1.03–1.36)^*^	1.26(1.04–1.53)^***^	1.21 (0.89–1.65) ^NS^
Holding a high school diploma	1.09(0.99–1.19) ^NS^	1.01 (0.91–1.12) ^NS^	0.99 (0.88–1.13) ^NS^	1.11 (0.91–1.34)^***^	1.12(0.80–1.56) ^NS^
University	Ref	Ref	Ref	Ref	Ref
**activity status**					
seeking work	0.72(0.52–0.98)^*^	0.83(0.59–1.16) ^NS^	0.60 (0.36–1.00) ^*^	1.31 (0.74–2.32)^***^	0.84 (0.42–1.70) ^NS^
Student	2.19(1.33–3.61)^***^	2.80(1.59–4.92)^***^	3.24 (1.67–6.30)^***^	3.25(0.88–11.97)^***^	0.92 (0.16–5.11) ^NS^
Income recipient	2.49(2.38–2.61)^***^	0.54(0.51–0.57)^***^	0.43(0.39–0.46)^***^	0.55 (0.50–0.62)^***^	0.82 (0.73–0.92)^***^
homemaker	1.50(1.43–1.58)^***^	1.47(1.38–1.56)^***^	1.52(1.39–1.66)^***^	1.48(1.32–1.66)^***^	1.43(1.28–1.60)^***^
Other	1.70(1.61–1.81)^***^	0.93(0.87–1.00)^*^	1.34 (1.19–1.51)^***^	0.88(0.78–1.00)^***^	0.94(0.84–1.05) ^NS^
Employed	Ref	Ref	Ref	Ref	Ref
**age (years*)***					
60–69	0.22(0.21–0.23)^***^	0.20(0.20–0.21)^***^	-	-	-
70–79	0.57(0.55–0.59)^***^	0.53(0.51–0.55)^***^	-	-	-
≥ 80	Ref	Ref	-	-	-
Constant	-	0.89(0.80–0.99)^*^	0.17(0.15–0.19)^***^	0.47(0.38–0.56)^***^	0.77(0.58–1.02) ^NS^
Pseudo R2	-	0.16	0.10	0.15	0.12

^NS^means not significant, and Ref. represents the reference group

^*^displays significance at the 0.05 level

^**^at the 0.01 level

^***^at the 0.001 level

According to column 2 (Table 3), City residents are less likely to live alone than villagers, and women are more likely to live alone than men. The odds of living solo among illiterate and primary school elderly are 2.8 and 1.4 times higher than those with a university education.

Being seeking work, 28% decreased likelihood of solo living than being employed. The odds of living alone among students, pensioners, homemakers, and the elderly doing other activities are, respectively, 2.19, 2.49, 1.5, and 1.7 times more than elderly with employment history. Moreover, the odds of solo living of the elderly in the early and middle stages of old age is less than in late old age.

Based on column 3 (Table 3), in an adjusted model, after considering the effect of all variables, living in rural areas, being a woman, and old age still increases the likelihood of living alone. There is a reduction in the odds of solo living, as the level of education increases to the university level (except for Holding a high school diploma). Some changes are observed after controlling for other variables, the status of seeking work lost significance, and the effect of Income recipient and other activities was reversed. It is noteworthy that 16% of the variation in solo living can be explained by the changes in place of residence, sex, age, education, and activity status of the elderly (last row of Table 3).

Columns 4–6 (Table 3) display the controlled odds ratio for the early old age, middle old, and late old age. In early old age, urban solo living is higher than rural ones, but in middle age and late old age, urban solo living is lower than rural ones. In the three age groups, solo living decreases in the unemployed elderly and increases in the illiterate and primary educated elderly.

## Discussion

Examining the prevalence of living alone of the elderly during the five censuses nationwide showed that the number of elderly living alone is increasing in urban and rural areas. The proportion of people living alone increases with age, from the stages of early old age to middle old age and through late old age. The increase in the solitary life of the elderly is observed while the elderly and their parents had high fertility rates. Increasing the age of marriage and delaying childbearing reduces the impact of the decline of extended families. It also increases the years of presence of children in the paternal family [12].

Influenced by the norms of private life [5], which is a consequence of the modernization era, and the pressures of urban life, the elderly often prefer to have independent living arrangements to avoid disruption in their relationships with their children [12].

A comparison of older males and females living alone shows that the increase in the proportion of older people living alone is due to the increase in women. It is noteworthy that in all the census years, the proportion of women living alone was higher than that of men. This difference is mainly due to differences in marital behaviour and significant differences in the ratio of remarriages after the death or divorce of the spouse [5]. The most important social factor is the tendency of children to be at a distance and live independently of elderly parents, even after the death of their fathers [13]. Also, due to the differences in life expectancy between women and men, the number of older men living alone is lower than women [33]. In addition, women tend to live alone compared with men. They are also under the pressure of socio-demographic forces, which compels them to undergo such life conditions [12].

As shown in the study of Nazban et al., Villagers have more solitary life than the urban elderly [25]. One of the most common causes of solo living in rural areas is migration [34]. The migration of children causes changes in the structure and function of the family. Due to the alternative nature of migrations (mostly young population) [35] and the flow of migration (mostly rural–urban) [36], it has caused the spatial separation of parents from their children in many rural areas. These migrations can be managed, prevented, and reduced. Therefore, urban planners and policymakers must consider the balanced development of the different areas, including small towns and villages, to diminish the solo living of the elderly.

In this study, low education increases the likelihood of living alone, but in other studies, education has been considered a factor in increasing solo living [4, 6, 7], and in Wonder's study, there was no evidence that lower education increases the risk of solo living [8].

The study of activity status showed that being a student and homemaker increases solo living. Women are more homemakers than men, enabling them to maintain their independence and live alone according to the norms of private life [5].

In most developing countries, governments and policymakers tend to value the family system as a source of support for the elderly, but due to weak informal and familial support, there is a need to strengthen social support networks in old age. It is noteworthy that the Iranian elderly do not live alone because there is no formal nursing home but prefer to live alone due to the negative propaganda against the nursing home. Therefore, creating a culture is necessary to eliminate this pessimistic view.

The strength of this study was the large sample size and the quality of the data collected. The main limitation was the cross-sectional nature of the study design, which limited the causal inference of solo living patterns over time. Covariates that explain the potential risk factors for living alone in the elderly, such as health status, disability, or chronic illness due to secondary data analysis, were not available.

## Conclusions

Despite the limitations, this study provides a significant contribution to the existing literature on the socio-economic factors related to the solidarity life of the elderly in Iran. The results showed that the prevalence of the elderly living alone is increasing. Also, the prevalence of solo living is higher among the elderly living in the eastern provinces of Iran. From this study, it can be concluded that the odds of solo living is higher in late older age, in women, in people who have lower educational level, who live in rural, in student and homemakers. In addition, knowing about the trend and characteristics of the elderly living alone will guide both the family and the public and private sectors on issues that should be given priority.

## Data Availability

The data that support the findings of this study are available in the statistical centre of Iran at: https://www.amar.org.ir/english/Population-and-Housing-Censuses.
